# Exposure to hycanthone alters chromatin structure around specific gene functions and specific repeats in *Schistosoma mansoni*

**DOI:** 10.3389/fgene.2014.00207

**Published:** 2014-07-16

**Authors:** David Roquis, Julie M. J. Lepesant, Emanuel Villafan, Jérôme Boissier, Cristina Vieira, Céline Cosseau, Christoph Grunau

**Affiliations:** ^1^Département de Biologie, Université de Perpignan Via DomitiaPerpignan, France; ^2^CNRS, UMR 5244, Écologie et Évolution des Interactions (2EI)Perpignan, France; ^3^CNRS, UMR 5558, Laboratoire de Biométrie et Biologie Évolutive, Département de Biologie, Université Lyon 1Villeurbane, France; ^4^Red de Estudios Moleculares Avanzados, Instituto de Ecología A.C.Xalapa, México; ^5^Département de Biologie, Institut Universitaire de FranceParis, France

**Keywords:** *Schistosoma mansoni*, resistance, induction, hycanthone, oxamniquine, epigenetics, repetitive sequences

## Abstract

*Schistosoma mansoni* is a parasitic plathyhelminth responsible for intestinal schistosomiasis (or bilharzia), a disease affecting 67 million people worldwide and causing an important economic burden. The schistosomicides hycanthone, and its later proxy oxamniquine, were widely used for treatments in endemic areas during the twentieth century. Recently, the mechanism of action, as well as the genetic origin of a stably and Mendelian inherited resistance for both drugs was elucidated in two strains. However, several observations suggested early on that alternative mechanisms might exist, by which resistance could be induced for these two drugs in sensitive lines of schistosomes. This induced resistance appeared rapidly, within the first generation, but was metastable (not stably inherited). Epigenetic inheritance could explain such a phenomenon and we therefore re-analyzed the historical data with our current knowledge of epigenetics. In addition, we performed new experiments such as ChIP-seq on hycanthone treated worms. We found distinct chromatin structure changes between sensitive worms and induced resistant worms from the same strain. No specific pathway was discovered, but genes in which chromatin structure modifications were observed are mostly associated with transport and catabolism, which makes sense in the context of the elimination of the drug. Specific differences were observed in the repetitive compartment of the genome. We finally describe what types of experiments are needed to understand the complexity of heritability that can be based on genetic and/or epigenetic mechanisms for drug resistance in schistosomes.

## Introduction

*Schistosoma mansoni*, a platyhelminth trematoda parasite, is the causing agent of intestinal schistosomiasis (or bilharzia). The parasite has a heteroxenous life cycle with two hosts and three main developmental stages. The cycle begins when eggs come in contact with fresh water and hatch, releasing a free-swimming larva known as miracidium. This larva infects the intermediate host (a freshwater snail, usually of the *Biomphalaria* genus). Inside the mollusk, it will transform into primary and then secondary sporocysts and undergo asexual multiplication. A second type of free-swimming larvae, cercariae, is released and infects the definitive mammalian host (human or rodent) in which they will reach their adult form (worm) and perform sexual reproduction. Sexually mature schistosomes couples are located in mesenteric veins of the mammalian host. Females lay eggs with a characteristic lateral spine, which allow eggs to go through the wall of the intestine and be excreted in the feces. However, a large proportion of eggs is caught in the blood circulation and end up being trapped in the liver, leading to hepatic fibrosis. Bilharzia is an endemic disease in many areas in Africa and South America, infecting 67 million people and causing an important socio-economical burden (King, 2010). Since the beginning of the twentieth century, several schistosomicides were developed (Galdino da Rocha Pitta et al., 2013). Many of them were abandoned due to low treatment efficiency, as well as toxic and sometimes mutagenic side effects to the human host (Galdino da Rocha Pitta et al., 2013). Another major concern regarding schistomicides is the rapid appearance of resistances, both in laboratory strains and natural populations of the parasite (Bruce et al., 1987; Coles et al., 1987; Drescher et al., 1993; WHO, 1998). Please note that to avoid any language confusion, we use here the definitions of drug resistance and tolerance in schistosomes as given by Coles (2002), with the former being “*when any isolate has a significantly lower cure rate than the most sensitive isolate*” and the later when the whole species is non-responsive to the drug. These factors left a single drug, praziquantel, as the current therapeutic alternative. As there is very little advance in the development of novel drugs against schistosomiasis, it becomes crucial to deepen our understanding on which molecular and evolutionary mechanisms can lead to schistosomicides resistance. Such knowledge could allow to either avoid the rapid appearance of resistances previously seen in *S. mansoni*, or to find ways to circumvent it.

Various drug resistances in schistosomes were studied through the past decades, but one of the best documented, but also most intriguing one, is against hycanthone. This molecule is not used anymore in treatments, and has been commercially discontinued, but was subjected to many research works from different laboratories. First evidences of resistant worms to this molecule were published in the early 1970's (Rogers and Bueding, 1971). It was later demonstrated several times that schistosomes were in general cross-resistant or cross-susceptible to both hycanthone and another schistosomicide, oxamniquine (Jansma et al., 1977; Cioli and Pica-Mattoccia, 1984; Dias and Olivier, 1985; Bruce et al., 1987). Oxamniquine was often used as a proxy for hycanthone, as the later is not commercially produced anymore and was toxic to the host to a certain degree. Two laboratories obtained stable resistant lines of schistosomes by injecting infected mice with a single curative dose (i.e., killing >90% of the worms in susceptible lines, 80 mg/kg) repeated over 3–5 successive generations of the parasite (Cioli and Pica-Mattoccia, 1984; Dias and Olivier, 1985). In these lines, resistance was heritable and stable even in the absence of drug pressure over 9–30 generations (Dias and Olivier, 1985; Cioli et al., 1989; Drescher et al., 1993). Classic genetic crosses between sensitive and resistant strains, as well as genetic complementation demonstrated that a single recessive autosomal locus codes for the hycanthone/oxamniquine resistance in two strains, MAP (named from the initials of the patient from which it was isolated) and Baltimore Rome Resistant (BRR) (Cioli and Pica-Mattoccia, 1984; Cioli et al., 1989, 1992; Pica-Mattoccia et al., 1992b, 1993). Similar results were also obtained *in vitro* by Coles and Bruce (1987). They cultured schistosomulas (an immature developmental stage occurring once cercariae have entered the mammalian host) under 240 p.p.m. of oxamniquine, a lethal concentration for 99.8% of the population. Survivors were injected in mice, and their progeny was not responsive to very high concentrations (500 mg/kg) of the drug. Experiments with tritiated hycanthone and oxamniquine demonstrated that the molecules were absorbed by all worms, but did not persist in resistant worms, while they were found to be covalently bound to DNA, RNA, and proteins in the sensitive strains (Pica-Mattoccia et al., 1988, 1989). Biochemical characterization showed that an enzymatic activation, probably by a sulfotransferase (Pica-Mattoccia et al., 2006), was needed by both oxamniquine and hycanthone to be lethal to the worm. Prior to its formal characterization, Pica-Mattoccia et al. (1992a) were able to biochemically isolate a fraction containing this enzyme from ground sensitive worms. They showed that resistant worms were killed when exposed *in vitro* to either hycanthone or oxamniquine and to the sulfotransferase-containing fraction from sensitive worms, while unaffected when exposed to one of the drugs alone. In the same article, the authors established that adult male worms, that are more sensitive to the drugs than females, have a higher enzymatic activity than females and immature stages (mostly unaffected by the drugs at the classical therapeutic concentration, and having a very low enzymatic activity). A difference in the enzyme specificity might also explain why hycanthone, but not oxamniquine, is efficient against *Schistosoma haematobium* (Pica-Mattoccia et al., 1997). The sequencing of the genome of *S. mansoni* (Berriman et al., 2009) provided new opportunities of investigation, and Valentim et al. (2013) recently used linkage mapping methods to identify the gene responsible for resistance in the HR and MAP strains. Candidate genes were screened using recombinant proteins, RNAi, and protein crystallography. The authors showed that it was indeed a sulfotransferase that was responsible for the resistance. They identified its coding gene (Smp_089320) and the non-synonymous nucleotide substitutions causing the loss of function of the enzyme in resistant lines MAP and HR, thus clearly establishing the mechanism of resistance in the studied lines (see Figure S1 in Valentim et al., 2013 for the mode of activation of oxamniquine).

Nevertheless, there are several observations that suggested early that alternative mechanisms might exist. For instance, Jansma et al. (1977) described three different ways to induce resistance by collecting the progeny of worms after a single injection of 60 mg/kg of hycanthone to the rodent host 54–70 days post infection (Type I), or 27–29 days post infection when worms are still at an immature stage (Type II), and from hosts infected by a single sex and then re-infected 2–58 weeks later by cercariae of the opposite sex (Type III). In all three cases, parents were all susceptible to the drug while a high percentage of the progeny survived the administration of a curative dose of hycanthone. Resistance was shown to be metastable to various degrees (depending on the type of induction used) through 10–21 generations. By metastable, we mean that from one generation to another, the percentage of worms surviving hycanthone treatment was highly fluctuant and did not follow a classical Mendelian inheritance pattern. For example, it was observed at several occasions by Jansma et al. (1977) that while a parental generation was completely resistant (100% survivors), a high proportion of the direct progeny (sometimes up to 75%) was found sensitive. Sometimes, the amount of resistant parasites would go up again a couple of generations later. These results are in complete contrast of the work mentioned above where classical recessive monogenic pattern for the inheritance of the resistance was observed. The work of Jansma was partly reproduced by Brindley et al. (1989). They were able to achieve Type II induction with lines of schistosomes deriving from Jansma's isolate.

As these observations do not fit the monogenic resistance model recently described, we reasoned that environmentally triggered drug resistance with metastable inheritance is a legitimate candidate for an epigenetically based phenotype. We use here a more narrow definition of epigenetics, meaning heritable changes in gene expression, without changes in the DNA sequence (Wu and Morris, 2001). They can be physically based on chemical modification of the DNA (e.g., methylation of cytosines), changes of proteins interacting with DNA (e.g., histone modifications), as well as nuclear localization of chromosomes, and short untranslated RNA playing a role in post-transcriptional silencing of genes (Aguilera et al., 2010). There are numerous examples in the literature on how environmental factors, such as nutrition, pollution, temperature, and others, can have an influence on epigenetic control (Feil and Fraga, 2012). A drug treatment could, for example, induce an epimutation, silencing the gene coding for the enzyme responsible of the bio-activation. This epimutation would be more or less stable and/or inherited to progeny given the parasite strain (as the phenotype was previously described to be either stable or metastable through generations depending on investigators and strains). This type of transient transgenerational modification was initially also called “Dauermodifikation” (Jollos, 1921).

From this hypothesis, we decided to revisit the historical data and to complement it with our own study. DNA methylation has been found to regulate oviposition (Geyer et al., 2011). However, it is globally at a very low level through the genome (Raddatz et al., 2013), and therefore we decided to focus on another epigenetic information carrier, histone modifications. We decided to use a Brazilian (SmBre) and Guadeloupian (SmGH2) strain. Both strains were previously pool-sequenced (Clément et al., 2013) and we found that they were both of low genetic diversity (Tajima's pi of 2.10^−4^ for SmGH2 and 1.8.10^−4^ for SmBRE) and do not have any coding polymorphism in the gene (Smp_089320) uncovered by Valentim et al. (2013). Even if there is an almost uniform genetic background within the strains, we could not, at this stage completely rule out that an induction phenotype could come from the selection of a novel resistance allele in some schistosomes and not an epigenetic change. The debate whether heritable characters are induced by the environment or whether they occur randomly and are subsequently selected is not new. In 1951, in order to differentiate between “spontaneous (genetic) mutation and natural selection” and “directed mutation” or induction, the Lederbergs had designed their now famous experiment using replica plating of bacterial colonies from non-selective support to Petri dishes with selective media (Lederberg and Lederberg, 1952). Their work gave results in favor of the spontaneous mutation, i.e., a genetic variability (diversity) based mechanism. We reasoned that it would be in principle possible to reproduce the classical Lederberg experiment, but this time using clonal populations of *S. mansoni* instead of bacterial clonal colonies.

Our results show that one of the strains, SmBre, is resistant to hycanthone in the absence of mutations in Smp_089320. After exposure to the drug, the chromatin structure is altered around genes belonging to gene ontology (GO) groups involved in catalytic activity and response to stress. This finding suggests that epigenetic mechanisms could be responsible for adjustments of the parasites metabolism leading to temporary resistance, thus reconciliating the seeming contradictions between stable and metastable types of the phenotype.

## Materials and methods

### Maintenance of the parasites

The *Schistosoma mansoni* strains SmBre and SmGH2 were originally sampled in Brazil and Guadeloupe, respectively. Both strains were maintained in their sympatric intermediate host strain BgBre and BgGua of the mollusk *Biomphalaria glabrata* and *Mus musculus* as definitive vertebrate host. SmBre was originally sampled in the 1960s in Recife, and SmGH2 was isolated on Nov. 10th, 1983 from a patient in Ste. Rose (Guadeloupe, French West Indies) (Pers. Commun. A. Théron).

### Preliminary drug screening of the strains

Both SmBre and SmGua were screened for resistant individuals by first infecting five mice (for each strain) with 70 mixed-sex cercariae. Mollusks from which cercariae were collected were initially infected with 20 miracidia each, and hence cercariae shed are of multiple genotypes. Mice were injected 62 days later with a dose of 60 mg/kg of hycanthone (similar to the one used by Jansma et al., 1977). Eight weeks later, the mice were sacrificed by a lethal intraperitoneal injection of sodium pentobarbital, and surviving adult worms were recovered by retrograde perfusions of the hepatic portal system with citrate (7.5%) saline (8.5%) solution administrated through the left ventricle (Duvall and DeWitt, 1967). Worms trapped in the liver or mesenteric system were collected after excising these organs.

### Lederberg-like experiment

**Figure 1:**
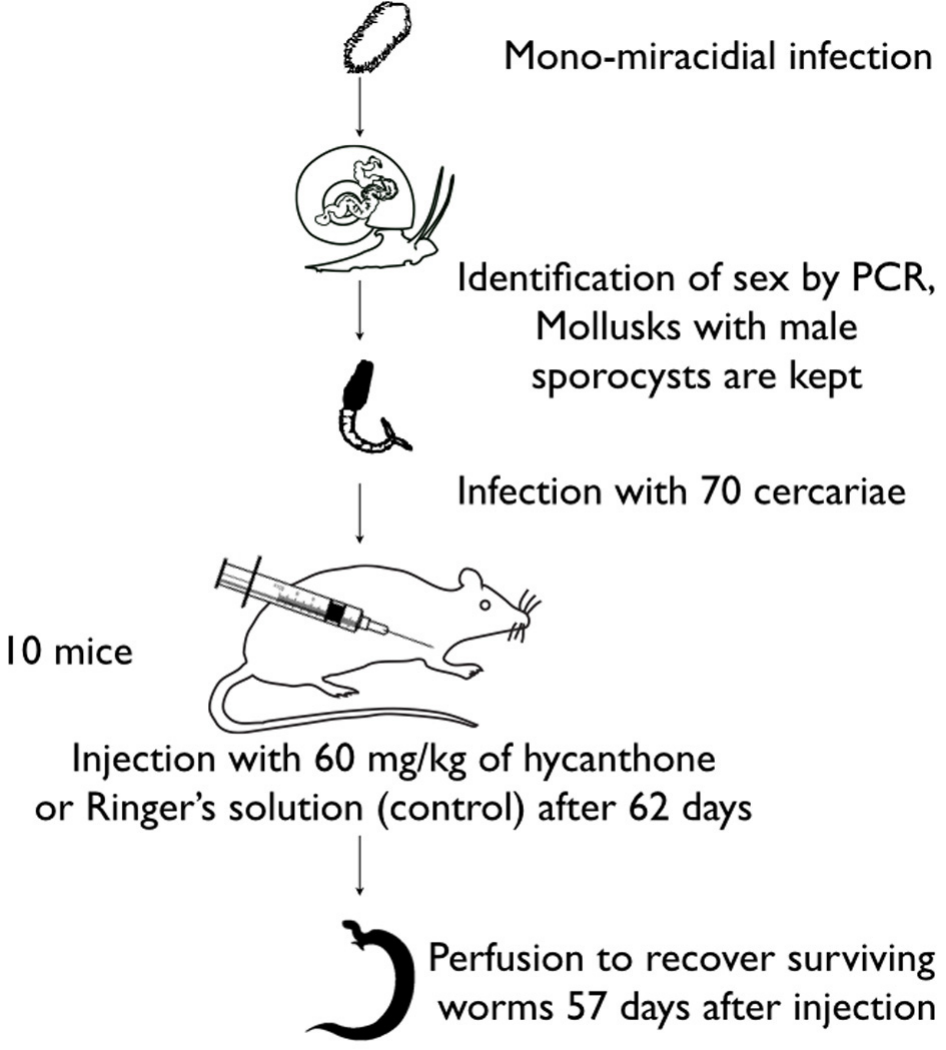
**Graphical abstract of the methodology used for the Lederberg-like experiment**.

*Biomphalaria glabrata* BgBre individuals were infected with single miracidium from SmBre. The experiment was repeated with several different *B. glabrata* BgBre host individuals, each one infected with a different *S. mansoni* clone (each miracidium came from different parents). We genotyped with sex markers cercariae shed by each *B. glabrata* following Beltran et al. (2008), and we selected five mollusks infected with male parasites. We chose to work only on male schistosomes as it has been shown that they are more sensitive to hycanthone (Pica-Mattoccia et al., 1992a) than females, and to avoid sex-based bias in the epigenome analysis. From each snail, we infected 10 mice with 70 cercariae each. Sixty-two days later, half of the mice were injected with a dose of 60 mg/kg of hycanthone, and the other half with Ringer's solution as a negative control. Fifty-seven days after injection, we perfused the mice following the method of Duvall and DeWitt (1967) to collect control and surviving, resistant worms. A graphical abstract of the methodology is on Figure 1.

### Chromatin immunoprecipitation and sequencing

Native ChIP was performed following the protocol developed for *Schistosoma mansoni* by Cosseau and Grunau (2011) (also available online at http://methdb.univ-perp.fr/cgrunau/methods/native_chip_sm.html) on a pool of six resistant and a pool of 10 control adult worms. Immunoprecipitation was performed using the following antibodies: H3K4me3 (Millipore, cat# 04-745 lot# NG1680351, 4 μ l per reaction), H3K9Ac (Millipore, cat# 07-352 lot# DAM16924924, 8 μ l per reaction), H3K9me3 (Abcam, cat# ab8898 lot# 733951, 4 μ l per reaction), and H3K27me3 (Diagenode, cat# pAb-069-050 lot# A29900242, 8 μ l per reaction). ChIP products were sequenced as single-end 50 bp reads on an Illumina HiSeq 2000 at the Montpellier GenomiX facility (http://mgx.cnrs.fr). We performed PCR amplification of the two exons of Smp_089320 on control and resistant ChIP products to test if a possible somatic mutation had occurred in that gene. We used Promega GoTaq Flex (cat# M8291) at 3 mM MgCl_2_ and with the following PCR conditions: initial denaturation 2 min at 95°C followed by 35 cycles of denaturation 95°C for 30 s, annealing 62°C for 30 s and elongation 72°C for 1 min. A final extension of 72°C for 5 min completed the reaction. We used two primer pairs to amplify exon 1 (Fwd1 tccacctctctctcactcaatg, Rev 1 ccacatgttggtaaatccgta, Fwd2 gctggtctaccgagaactgg and Rev2 tcccgtagaaaaccaactcg) and one pair for exon 2 (Fwd agtccattcattcaatgtttcaa and Rev caatccacaaatccccattc). PCR products were gel purified using Promega Wizard SV Gel and PCR Clean-Up System (cat# A9282) and sequenced at the Genoscreen facility (http://www.genoscreen.fr/).

### Quality control and alignement

All data treatment was carried out under a local galaxy instance (Goecks et al., 2010) (http://galaxy.univ-perp.fr). We used the FASTX-toolkit (http://hannonlab.cshl.edu/fastx_toolkit/) for verification of the reads quality. Read quality was judged sufficiently good (the majority of reads showed a fastqsanger quality score ≥24 for all positions). For the analysis of unique sequences, reads were aligned on the *Schistosoma mansoni* genome assembly v5.0 (Protasio et al., 2012) with Bowtie2 (Langmead and Salzberg, 2012) using parameters—sensitive -k 2. Reads with unique match were filtered with samtools (Li et al., 2009) (samtools view -hS -q 255). For peak calling, an equal number of randomly selected reads was chosen between the treated and control conditions (H3K4me3 = 2.9 million, H3K9Ac = 23 million, H3K9me3 = 21 million, H3K27me3 = 17.9 million). Peak identification was performed using PeakRanger v1.16 (Feng et al., 2011) with *P*-value cut off 0.0001, FDR cut off 0.01, Read extension length 200, Smoothing bandwidth 99 and Delta 1. Wiggle files generated by PeakRanger where uploaded in our local instance of GBrowse (Stein et al., 2002) (http://genome.univ-perp.fr) for visualization. For the analysis of repetitive sequences, reads were aligned with Bowtie2 evoking parameters—sensitive -k 5. The 3,145 repetitive consensus sequences used as reference in this study were obtained from a previous analysis done with the same strain SmBre (Lepesant et al., 2012). From the resulting SAM file, we choose the position with the highest score for each read. Normalization of SAM files was done by randomly removing a given portion of the counts to achieve the same effective library size (H3K4me3 = 0.7 million, H3K9Ac = 9.8 million, H3K9me3 = 10.8 million, H3K27me3 = 13.9 million). Elements that showed differences in histone modification levels where annotated using the ABBlast search engine within the RepeatMasker program (Smit et al., 2010). The search was performed for both nucleotide and protein databases. Sequences without match where compared against the nucleotide database of NCBI using the blastn search engine and against the non-redundant protein database using blastx (Altschul et al., 1990).

### Comparative analysis

To find differences in the histone mark pattern between control and treated conditions, we used the Pyicoenrich function (with –binsize 0.3) from Pyicos v2.0.6 (Althammer et al., 2011). Visual inspection was performed on all the regions detected by the software with a z-score above 7. The GO enrichment analysis was performed using BLAST2GO (Conesa et al., 2005). For the analysis of significant differences in histone isoform enrichment in repeats, normalized read counts were compared using the DESeq package of the R software (Anders and Huber, 2010). This package uses the negative binomial distribution to calculate the *P*-value for each element between two compared samples and find elements that present significant differences in their enrichment levels.

### GO enrichment analysis

The 60 genes for which chromatin structure differences had been detected were annotated using Blast2GO (Conesa et al., 2005). In order to check if GO terms would be overrepresented in this group of genes, 60 transcripts were randomly sampled out of 41,669 transcripts generated by a combination of RNA-Seq data and Sanger annotation. RNA-Seq data were generated from several biological replicates and these data will become available when the analysis is fully finished. The random samples were annotated as above and GO enrichment analysis was performed using Fisher's exact test implemented in Blast2GO and a *p*-value of 0.2 as cutoff. Random sampling was repeated 3 times.

## Results

### A strain with sensitive genotype displays hycanthone resistance

We compared the number of worms recovered in mice injected with hycanthone to those recovered from control mice. We found 25 (36%) surviving worms, perfused from 5 mice, in the Brazilian strain SmBre, despite the fact that it has the sensitive genotype for Smp_089320. The Guadeloupian strain SmGH2, however, is completely sensitive to hycanthone, i.e., no survivors were found in any of the five infected and treated mice.

### Clonal schistosomes respond differently to hycanthone treatment

**Figure 2:**
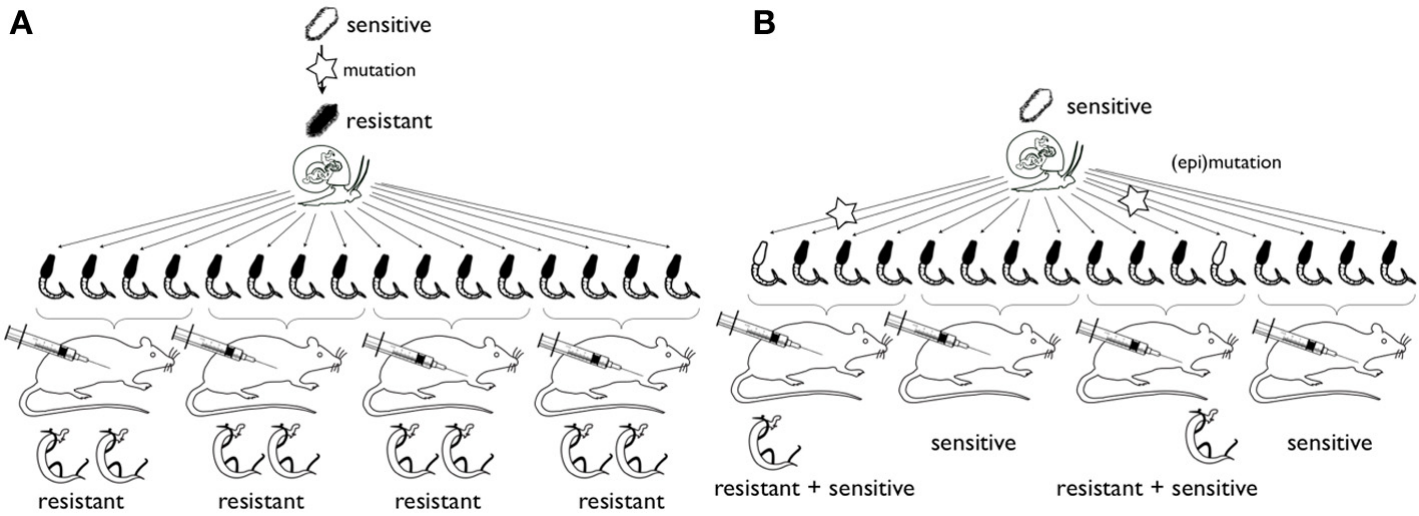
**Schematic representation of the experimental strategy for study of the origin of hycanthone resistance**. Hypothesis **(A)** rare mutations (star) in miracidia (top) confer resistance to hycanthone. All offspring will be resistant (or sensitive, not shown). Hypothesis **(B)** epimutations or mutations occur during clonal amplification of cercaria in the snail host. Only few adult worms will be resistant. In the case of epimutation, in subsequent generations reversal of the phenotype would occur (and was described in the past) and a chromatin structure changes can be detected. In case of mutation, no reversal occurs and mutations should be detected in the locus of interest.

**Figure 3:**
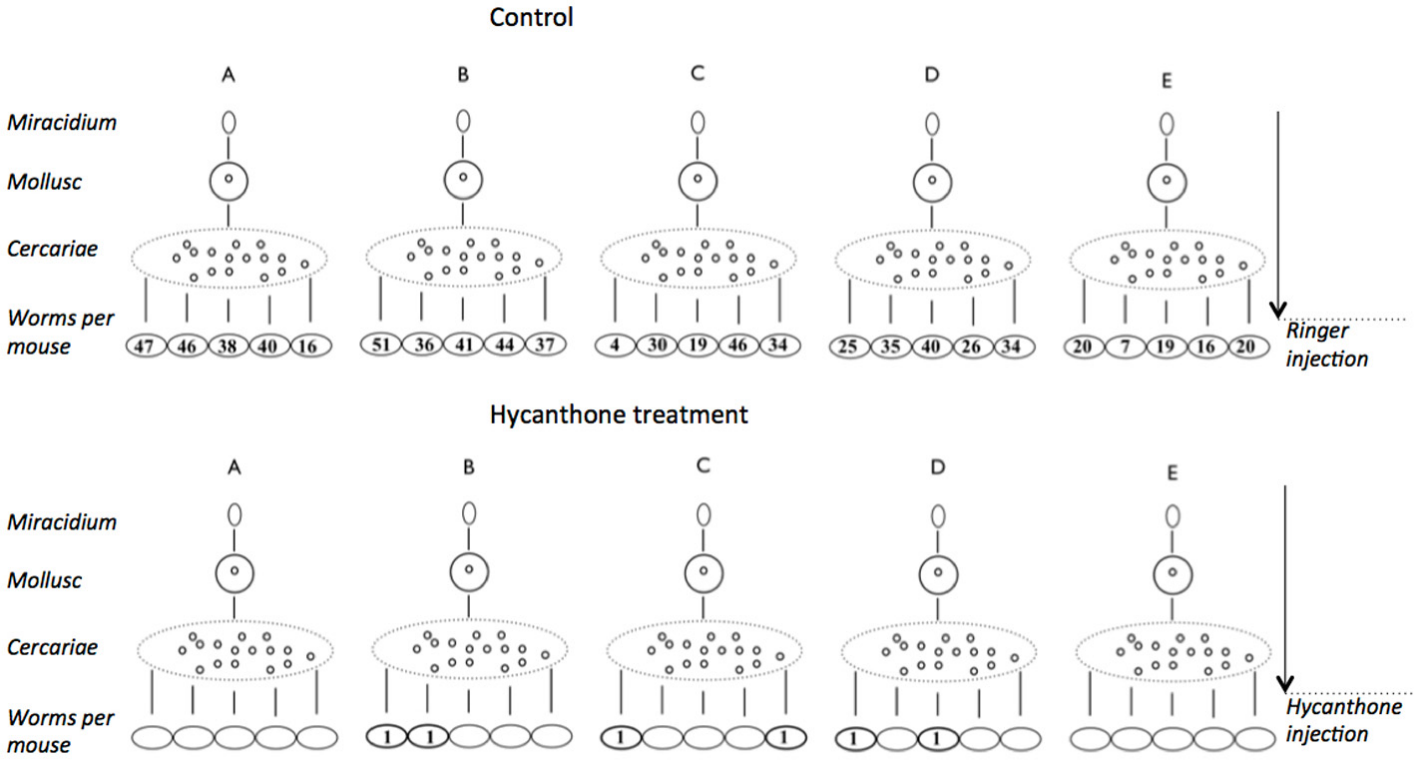
**Results of the experiment on induction of hycanthone resistance using mono-miracidial infections**. Five mollusks were infected using a single miracidium (genotypes A–E). Cercariae shed from a single mollusk are clonal and were used to infect 10 mice. Half of the mice were injected with a control Ringer's solution and the other half with a therapeutic dose of hycanthone. Mice were later perfused and worms were counted. In the hycanthone condition, six worms of three genotypes (B–D) were recovered from six mice, while an average of 30 worms per mouse were found in the control condition.

Following the discovery of hycanthone resistant individuals within the SmBre population, we adapted the Lederbergs' experiment to our schistosome model. To do so, we infected five *Biomphalaria glabrata* with a single, genetically distinct, SmBre miracidia. Cercariae shed by each mollusk are genetically identical. We then infected 10 mice with 70 cercariae. Half of the mice were then treated with an injection of hycanthone similar to the one described by Jansma for Type I induction (Jansma et al., 1977), and the other half was injected with Ringer's solution, as negative controls. As we excluded the possibility of a somatic mutation in Smp_089320, similar to the classical replica plating experiment, two possible outcomes can be imagined: (1) if a random mutation had occurred in one of the clones that conferred resistance to hycanthone (pre-existence), then only cercariae from this very snail would produce resistant flukes because they are genetically identical (Figure 2A) (2) if the phenotype is induced through the treatment with a certain probability, we would observe resistance randomly associated with different mice hosts and without correlation with the particular snail host (similar to Lederbergs' bacterial colony, Figure 2B). We later perfused the mice to look for control and surviving, resistant worms. The results of our experiment are shown in Figure 3. In summary, while the vast majority of mice were exempt of parasites after hycanthone injection, we found surviving worms in six different rodents, belonging to three different *S. mansoni* genotypes (B–D). We did not find any worm originating from genotype A and E after the treatment. In contrast, under the control condition, we recovered an average of 30 ± 5 worms (confidence interval at α 5%) per mice for the five genotypes. Sequencing of Smp_089320 in resistant worms did not show any of the three mutations identified by Valentim et al. (2013) or any new polymorphism.

### Chromatin structure changes associated with the resistant phenotype

We performed ChIP-Seq on the pool of the six hycanthone resistant worms, as well as on control worms. We targeted four modifications of histone H3: tri-methylation of lysine 4 [H3K4me3], tri-acetylation of lysine 9 [H3K9ac], tri-methylation of lysine 9 [H3K9me3], and tri-methylation of lysine 27 [H3K27me3]. The first two are associated with euchromatin (relaxed DNA structure allowing transcription) and the later two are associated with heterochromatin (condensed DNA structure impeding transcription). Around Smp_089320 none of the marks showed enrichment. We then searched for variations in the chromatin structure at the genome level, and a bioinformatic analysis, followed by visual inspection, was able to detect differences in the histone modification profiles between hycanthone resistant and control worms. We found 47 differences for H3K4me3, 43 of which being located at the transcription start site of genes. This corresponds roughly to 0.6% of the total peaks detected for this histone mark. For H3K9me3, 12 differences were spotted (0.012% of total peaks), mostly in introns and within 2 kb upstream and downstream of genes. We identified five differences for each of the other two marks (0.007% and 0.005% of total peaks for H3K9Ac and H3K27me3, respectively), the majority being outside of genes. A list of the regions and associated genes in which we found differences is in Supplementary Table 1.

### Chromatin structure modifications are preferentially associated with genes related to catalytic activity

None of the genes in which differences were uncovered code for a sulfotransferase. Using a GO enrichment analysis, we did not find any clear pathway linking together the genes bearing chromatin structure changes. However, we found that the terms “transferase activity” (13 occurrences), “kinase activity” (8 occurrences) and “catalytic activity” (26 occurrences) and “response to stress” (5 occurrences) were overrepresented in the panel of genes with epimutations.

### Chromatin structure modifications in repetitive sequences

We identified, in 14 repetitive sequences, statistically significant changes in chromatin structure. This is very few (0.44% of repeats) showing that globally the chromatin remains stable. Interestingly, changes in H3K4me3 and H3K9ac always decrease in the treated worms while H3K27me3 increases, indicating a tendency toward heterochromatization. No changes occur in H3K9me3. H3K4me/H3K9ac modifications are observed in dispersed repeats that belong to the LINE/LTR classes. In contrast, H3K27me3 becomes enriched in simple repeats that are organized in blocks (Supplementary Table 2) sometimes covering the entire length of large contigs for which chromosomal location is unknown.

## Discussion

When screening the literature on hycanthone resistance, we were surprised to see such divergent results between the different research groups. On one hand, there is clearly established monogenic, stably inherited, resistance, and on the other, there is an inducible resistance, appearing from sensitive parents, and not inherited in a Mendelian fashion. We took a deeper look at the methodology employed by the all the research groups, and their interpretation of data, with the hope of finding variations in the procedures, or in the analysis methods, that could, at least partly, explain the discrepancies between the observations and conclusions.

We first tried to track back the origins of the strains that were employed in the various studies on hycanthone/oxamniquine resistance (Table 1). Our conclusion is that very few strains were used in these studies, and most of them had been maintained in laboratory for a very long time, probably reducing the genetic diversity of the strains. We identified six resistant strains in the literature, and four of them derived from the M strain used in Jansma et al. (1977) for the first induction experiments. We also found four main sensitive strains. At least four other strains (either resistant or sensitive) were punctually used and will not be discussed here (Bruce et al., 1987; Drescher et al., 1993; Pica-Mattoccia et al., 1993).

**Table 1:** **Origin and resistance phenotypes of the most commonly used strains in the literature**.

**Strain**	**Origin**	**Resistance**	**Derived from?**	**Comment**	**References**
M	Puerto Rico	Oxa/Hyc	–	Used for the three types of induction experiments	Jansma et al., 1977
BRR	Puerto Rico	Oxa/Hyc	M	Underwent drug selective pressure over 3 generations	Cioli et al., 1992; Pica-Mattoccia et al., 1992a,b, 1993, 2006
JHU	Puerto Rico	Oxa/Hyc	M	Successful Type II induction	Brindley and Sher, 1987; Brindley et al., 1989, 1991
H-30	Puerto Rico	Oxa/Hyc	M		Souza et al., 1988
HR	Puerto Rico	Oxa/Hyc	M		Valentim et al., 2013
MAP	Brazil, Minas Gerai	Oxa Partial to Hyc	–		Dias and Olivier, 1985; Bruce et al., 1987; Souza et al., 1988; Pica-Mattoccia et al., 1992a,b, 1993; Drescher et al., 1993; Valentim et al., 2013
SEN	Puerto Rico	None	–		Cioli, 1976
BH	Brazil, Minas Gerai	None	–	Isolated from an untreated patient in 1967	Dias and Olivier, 1986; Bruce et al., 1987
NMRI	Puerto Rico	None	–	Isolated in the 1940'	Fletcher et al., 1981; Brindley and Sher, 1987; Brindley et al., 1989
LE	Brazil	None	–		Valentim et al., 2013

Hyc, Hycanthone resistant; Oxa, Oxamniquine resistant.

In his Type I induction, Jansma (Jansma et al., 1977) injected a dose of 60 mg/kg of hycanthone 54–70 days post-cercarial infection. He noted a marked reduction in the number of worms, but rarely a complete elimination. Surviving worms were unable to produce viable eggs for 4–8 months. After that period, eggs from which miracidia were hatched were collected to produce a F1. This first generation was treated with a therapeutic dose of hycanthone (80 mg/kg) and 94% of the worms were found to be resistant. Twenty-one successive generations were produced from this F1, and on several occasions, additional groups of mice were infected from cercariae batches shed on different days, by the same infected mollusks, leading to parallel generations known as branches. Resistance tends to decrease with generations in the main branch, and is highly variable between branches (some are totally resistant for several generations, some become completely sensitive, and others are fluctuant). Although it is not explicitly stated in the paper, it seems that progeny for generations F2–F21 was collected before the therapeutic drug injection used to estimate the percentage of resistant parent worms. Hence, there was no drug selective pressure from one generation to another, and that could partly explain the metastability observed by Jansma but not by other investigators, as suggested by Cioli and Pica-Mattoccia (1984). It is conceivable that Type I resistance was achieved not by induction, but through the selection worms with a resistant genotype, and as the induction dose is slightly below the therapeutic one (normally killing >90% of the parasites), there is possibility that some worms with a normally “sensitive” phenotype, escaped the drug. Cioli et al. (1992) observed that their resistant strain derived from Jansma's had a lower global infectivity (to mollusk and mammalian host) and a lower fertility (lower amount of eggs per females) than their sensitive strain (although this could be strain related, as the sensitive strain from Cioli is not from the same origin as Jansma's). In the hypothesis that some “sensitive” worms survived the induction step and were mixed with selected resistant worms, this could explain why the resistance is does not follow a Mendelian inheritance. This cannot be the case in Type II, as immature worms have little or no sensitivity to hycanthone (induction was also successful with sub-curative doses ranging from 3 to 60 mg/kg) and in Type III where the parents were not exposed to the drug before collecting the progeny. These observations lead us to think that the resistant phenotype in Type II and III could indeed be induced and transmitted to the progeny to a certain extent. Cioli and Pica-Mattoccia (1984), Cioli et al. (1989) and Dias and Olivier (1986) stated that they were unable to reproduce Jansma's results in inducing Type II or III resistance. Three other antihelminthics, oxamniquine, praziquantel, and oltipraz were also unsuccessfully tested with the hope of inducing Type II resistance and selecting a new line of resistant schistosomes (Dias and Olivier, 1986). However, we have a slightly different interpretation of the results from Dias and Oliver. In their induction protocol, mice were treated with subcurative dose of one of the three drugs, 26–30 days after cercarial infection (when schistosomula have a low sensitivity to both hycanthone and oxamniquine), and then divided into two groups. The first group was treated with a curative dose of drug, while the second was left as a control, and the eggs from that control group were used to restart a cycle of the parasite. Mice infected by the F1 cercariae from the control group were also divided into a trial group treated with a curative dose of the drug and a control group (which would serve to generate a F2). No resistant worms were found in the treated F1 and F2, but 8–12% where found in the parental generation (in which the induction step was performed). Admittedly, there was no inheritance of a resistance phenotype to F1 and F2, but the fact that survivors were found only in the parental generation which underwent the induction step let us believe that it was not as a failure at inducing resistance as described by the authors. In fact, only when induction was performed, some resistant worms were found. Type II was also successfully induced by the Brindley group (Brindley et al., 1989) in several lines of the parasite deriving from Jansma's isolate. However, while four strains tested in this article were described as sensitive by the authors, it is worth to mention that in some of the trials, up to 26% of the worms survived the hycanthone treatment (with or without preliminary Type II induction). Brindley also observed spontaneous occurrence of resistance in other strains with no induction, although he admitted that “*whether these fluctuations reflect true changes in the parasite genotype rather than experimental variation was not systematically investigated in this study*” (Brindley et al., 1989). This lead us to realize that for the majority of research groups, resistance was considered as achieved when at least 90% of the worm population survived a therapeutic dose. In several studies (Dias and Olivier, 1986; Brindley and Sher, 1987; Brindley et al., 1989; Drescher et al., 1993), various lines of schistosomes did not reach this resistance threshold (with or without induction and/or drug pressure over a couple of generations). They were considered as sensitive although in some cases there was a non-negligible percentage (3–26%) of worms surviving a therapeutic dose of either hycanthone or oxamniquine, strengthening our hypothesis that a mechanism following a non-Mendelian inheritance pattern is involved. In this context, we believe it is more appropriate to talk about the penetrance of the phenotype rather than talking strictly about sensitivity or resistance.

Also, Cioli and Pica-Mattoccia specifically mentioned that their approach was different from the Jansma/Bueding/Brindley group. Three generations of selection were necessary to obtain a phenotype that was stably inherited by the progeny (Cioli and Pica-Mattoccia, 1984). The same observation was made by Dias and Olivier (1985), as in their experiments, resistance was stable only after the 5th generation (experiments were continued until 14th generation). Unfortunately, the data for the first 3–5 generations are not available but one can assume that incomplete penetrance of the phenotype was observed. These large differences in penetrance of resistance phenotypes (i.e., large dose dependency) in different isolates were also found by others (Coles et al., 1986; Kinoti, 1987) and also described in Pica-Mattoccia et al. (1993). Kinoti noted important variations in the dose of oxamniquine needed to eliminate 99% of the worms (ED99), in both laboratory and clinical trials. For example, East African isolates needed a dose of oxamniquine 200–250% higher than Puerto-Rican isolates to reach ED99 (Kinoti, 1987). In all publications, at least 3 generations of selection were necessary to produce stable resistance, but even then, phenotypic differences between the different resistance strains and most notably an exception to the Mendelian segregation pattern were observed. In the MAP strain for instance there was (1) a difference between heritability of hycanthone and oxamniquine resistance and (2) segregation did not follow the recessive pattern. Segregation could not be followed up because the females produced no eggs, i.e., no genetic analysis was possible (“*Thus, we are left with no obvious explanation for this unexpected partial exception to the overall recessivity of the MAP resistance*.”) (Pica-Mattoccia et al., 1993). The reason for the incomplete penetrance in the first 3–5 generations cannot be determined with confidence from the published data alone. It might be that the initial strains showed a high degree of genetic diversity with only a few individuals carrying the mutation in the sulfotransferase gene. Molecular biology methods in *S. mansoni* research were not sufficiently advanced in that time to measure genetic diversity. From the few available data, however, it seems that the founder populations of the resistant strains were very small. Years of maintenance cycle in laboratories, as well as the possibility of a founder effect every time that the strains were transferred or shared with other laboratories let us think that the genetic diversity was probably low in the different strains. We also noted that most of the strains were from the same geographical areas (either Puerto Rico or the region of Minas Gerais in Brazil) and represent only a small share of geographic diversity of *S. mansoni.* Another reason for variability in penetrance could therefore be high phenotypic plasticity and/or variability in plasticity. Jansma's results were in favor of this latter mechanism, and he additionally described transgenerational plastic effects. It has also to be noted that while the work of Brindley and Jansma was performed using hycanthone, other research groups used oxamniquine. Although schistosomes where always cross resistant or cross sensitive to both molecules, hycanthone is known to have a mutagenic effect, and it could have played a role in the phenomenon of induction, which was never observed with oxamniquine.

In our own experiments, we worked with a strain bearing the sensitive genotype for the sulfotransferase gene previously identified as the source of hycanthone/oxamniquine resistance, but we still found in drug screening that in one strain (SmBre) about a third of the adult worms survived the treatment while another strain did not show survivors. Resistance of SmBre could be due to a mutation in a different locus and/or due to epimutations but this would still not explain Jansma's induction phenomenon. Therefore, we used a Lederbergs experiment to discriminate if the resistance was pre-existing or induced by the experimental conditions. Similar to the classical replica plating experiment, two possible outcomes could be imagined: (1) if the phenotype is induced through the treatment with a certain probability, we would observe resistance randomly associated with different mice hosts and without correlation with the particular snail host (similar to Lederbergs' bacterial colony) (2) if a random mutation had occurred in one of the clones that conferred resistance to hycanthone (pre-existence), then only cercariae from this very snail would produce resistant flukes because they are genetically identical. A caveat with the Lederberg experiment in organisms with epigenetic inheritance systems is the potentially heritable transcriptional and/or posttranscriptional level of diversity. It is possible that epimutations occur during clonal amplification of the cercariae (Figure 2B). Also, as long as somatic mutations cannot be excluded, mutation would do the same job. Both would mimic the outcome of the “induction” alternative. We found that not all the worms issued from the same miracidia (i.e., being genetic clones of each other), responded to the treatment the same way. While the vast majority was sensitive, we still found some resistant individuals. when interpreted in the context of Lederberg's experiment, would favor the directed (epi)mutation hypothesis. If we had selection of a pre-existing mutation or resistance phenotype, then we would have expected that all, or no, worms from a same genotype would present the same phenotype (resistance or sensitivity). From our results, we propose the hypothesis that epigenetics could be part of the mechanism underlying the resistance induction.

Our ChIP-Seq experiment provided evidences of epigenetic variation between resistant and control worms. Our analysis highlighted at least 64 chromatin structure changes between the two conditions, with an enrichment profiles in genes implicated in catabolic, detoxification, and transfer activity (Two examples can be seen in Supplementary Figure 1). No specific pathway linking these genes together arose, but it makes sense in the context of a response and the subsequent elimination to a drug to see these kinds of biological processes being involved (as stated before, hycanthone is known to be eliminated from resistant worms a certain amount of time after its penetration, while it is stuck in sensitive worms metabolism). It is therefore conceivable that the drug resistance observed in the surviving worms is due to epistatic interaction of multiple gene products rather than an (epi)mutation in a single locus. Another interesting finding is the presence of a differential H3K27me3 profile in the gene body of SmMRP1 (Multidrug Resistance Associated Protein 1, Smp_171740). In various species, including trematoda, this gene is part of a family of transporters playing a role in exclusion and elimination of xenobiotic compounds and metabolic toxins (Kasinathan et al., 2010). In schistosomes, it was demonstrated that SmMRP1 has higher levels of expressions in response to the widely used schistosomicide praziquantel, and possibly other drugs (Kasinathan et al., 2010; Greenberg, 2013). We found a significant enrichment in H3K27me3 around exon 21 of this SmMRP1 in the control condition. Enrichment of this mark are usually associated with increases of facultative heterochromatin and gene repression (Trojer and Reinberg, 2007; Bannister and Kouzarides, 2011). Our current knowledge of the functional role of histone modifications in gene expression in *S. mansoni* does not allow us to affirm that the lower presence of this mark in the resistant worms would lead to an increased expression of this gene, but it stays a promising candidate for further experiments.

Fourty-seven percent of the genome of *S. mansoni* is composed of repeats (Lepesant et al., 2012). The function of these repeats is completely unexplored and, in general, they are not included in the analysis of molecular basis of phenotypic variation. We have shown earlier (Lepesant et al., 2012), that repeats can be of importance in schistosomes, and we decided to study their chromatin structure changes upon hycanthone treatment. Only a very small fraction of the repeats (0.4%) change their histone modification status. In contrast to other organisms, stress (in this case hycanthone treatment) does not seem to lead to an euchromatization and potentially mobilization of repeats but has the inverse effect (decrease of H3K9ac and increase in H3K27me3). Interestingly, the repeats in which H3K27me3 status changes are located in satellite-type blocks on specific locations on the genome. It is conceivable that they form knobs that separate genome domains and influence indirectly gene expression. Further work will be needed to understand the impact of these repeated regions on the definition of the chromatin structure in which they are included.

While this ChIP-Seq experiment brings new insight on epigenetic mechanisms and regulation in *S. mansoni*, it does have some limitations. In order to be as faithful as possible to Jansma's work to reproduce his Type I induction, we exposed the worms to hycanthone through an injection in the rodent host. However, this generates a degree of variability from one mouse to another, as each individual has a different metabolism, immune system, clearance rate, and other physiological factor influencing the exposure of the worms to the drug. Also, although cercariae issued from a same initial miracidium are theoretically clonal, genomic instability such as somatic mutation and recombination can occur during asexual life stages like sporocysts, as shown by Vieira et al. (1991), and Bayne and Grevelding (2003), and their impact on the phenotype should be taken into account. Nonetheless, we managed to recover worms that survived the hycanthone treatment at two occasions (initial drug screening and Lederbergs-like experiment), from a low genetic diversity strain and without any evidences that the gene uncovered by Valentim et al. (2013) was involved. This, in addition with the differences of chromatin structure observed between surviving and control worms lead us to think that induction of resistance in *S. mansoni* is not a myth after all. Nevertheless, we do not think that induction should be seen as a specific response of the epigenome to a certain environmental condition leading to over/under-expression of a specific gene. Instead, this induction phenomenon seems to come from the capacity of some strains, or some individuals within a strain, to extend their degree of plasticity beyond the genetically defined “default” state through chromatin structure changes. This allows them to overcome an environmental stress in the absence of a pre-existing stress-response phenotype. Depending on the length of the stress, we can imagine that the progeny can more or less efficiently inherit the epigenetic condition responsible for the phenotype until it is fixed in the population (through a DNA mutation for example). This fits well with theoretical framework developed recently in which heritable phenotypic variation is based on at least two components: the low-fidelity (epigenetic) system and the high-fidelity (genetic) system (Klironomos et al., 2013). In this model, upon changes in the environment, the low-fidelity system can generate new phenotypes that can explore the adaptive landscape. The phenotype is decoupled from the genotype and appears before the adaptive genotype. The low fidelity of the epigenetic system means the adaptative phenotype could theoretically be loss as quickly as it appears, unless a strong selective pressure is applied. This is consistent with the observations of the metastability of hycanthone resistance in absence of drug pressure (Jansma et al., 1977; Brindley et al., 1989), and the stability of the phenotype after 3–5 successive generations of schistosomes where under drug pressure (Cioli and Pica-Mattoccia, 1984; Dias and Olivier, 1985). Basically, epigenetic variation allows to “buy time” for a few generation (in this case, 3–5) until a stable, genetically transmitted phenotype occurs. To understand better the underlying mechanism, measuring the expression level of Smp_089320 and of candidate genes identified here would be informative in future studies.

Since hycanthone is not anymore used for treatment of bilharzia and the use of oxamniquine is depreciated (Galdino da Rocha Pitta et al., 2013), mechanism of resistance is more of academic interest. However, experience with hycanthone and oxamniquine shows that a new conceptual framework is necessary to design experiments that distinguish the part of genetics and epigenetics in resistance phenotypes. We believe that a standard procedure would be beneficial for the testing of resistance to current drugs, especially praziquantel, the only molecule actually used for mass treatment of schistosomiasis. Resistance to praziquantel shares striking similarities with early observations on hycanthone/oxamniquine. It can be induced through subcurative, but increasing doses of praziquantel over six-seven generations, and genotyping of two populations of schistosomes showed that there was no significant difference in the genotypes before and after drug treatment, meaning no selection of specific genotypes by the drug (Blanton et al., 2011; Greenberg, 2013). We think that a standard procedure for the next drugs should be similar to the one we used to detect resistant individuals from a clonal population. Mono-miracidial infection would be a good start, as it minimizes the genetic variation between individuals and allows to detect changes in the epigenotype. A higher number of mono-miracidial infections, using several strains of *S. mansoni* (as the induction seems to be strain dependent), and a larger number of clones (adult or schistosomula) from each mono-miracidial infection should be tested, as we still do not have a clear idea of the frequency of the phenomenon. Induction step can be done *in vivo* (for any of the tree types) but screening of resistant progeny has to be performed *in vitro*, as described by Coles and Bruce (1987) and Pica-Mattoccia et al. (1992b). This is mandatory to make sure that all parasites have the same exposure to the drug. To look for heritability, resistant worms can be surgically reinserted into new rodent hosts and allow to reproduce. It is critical to have a standardized procedure to maintain the life cycle of the parasite (i.e., number of miracidia and cercariae, as well as their genotypes, used to infect the hosts) as it is difficult, if not impossible, in some older publications to find out if variation in the numbers of perfused worms come from effect of the drug or bias in infections. Using the same intermediate and final host is also important as they have an impact on the phenotype and life traits of the parasite. In the articles we reviewed, most experiments were done using the same final host (swiss albino mice), but sometimes hamsters were used, too. The geographical origin of the mollusk host is not always explicitly stated, and there are numerous evidences that it plays an important role in the parasite infection success and development (Theron et al., 1997). Although this was never explicitly discussed, it could be a cause to the contradictory results on hycanthone induction. Finally, both genomes and epigenomes of resistant worms would have to be compared to the ones of sensitive worms to figure out the origin of the phenomenon. Also, from our ChIP-Seq results, we saw that it is essential to keep a global, genome scale approach including repetitive sequences. The epimutations we found were distributed across the genome and are involved in various pathways. A candidate gene approach would probably have missed some of the variations we detected. Rather than only focusing on find a single gene responsible for the phenotype, we believe that the approach we describe would lead to a more complete answer on how schistosomes develop resistances.

## Ethic statement

The French Ministère de l'Agriculture et de la Pêche and French Ministère de l'Education Nationale de la Recherche et de la Technologie provided permit A 66040 to our laboratory for experiments on animals and certificate for animal experimentation (authorization 007083, decree 87–848 and 2012201-0008) for the experimenters. Housing, breeding and animal care followed the national ethical requirements.

## Author contributions

David Roquis performed the literature review, achieved the bioinformatics analyses and wrote the manuscript. Julie M. J. Lepesant designed and performed the experiments, contributed to prepare the reagents and the materials. Emanuel Villafan and Cristina Vieira analyzed the data concerning repetitive DNA. Céline Cosseau conceived the experiments and took part in writing the manuscript. Jérôme Boissier contributed to drug treatments and acquisition of samples. Christoph Grunau conceived and designed the experiments, contributed to analysis tool preparation and bioinformatics analyses, and took part in writing the manuscript.

### Conflict of interest statement

The authors declare that the research was conducted in the absence of any commercial or financial relationships that could be construed as a potential conflict of interest.
