# Supplementary material for: Exposure to hycanthone alters chromatin structure around specific gene functions and specific repeats in Schistosoma mansoni
Source: Front Genet. 2014 Jul 16;5:207. doi: 10.3389/fgene.2014.00207 (PMC4099960; doi:10.3389/fgene.2014.00207)
Supplement: Supplementary file 1 [file Presentation1.PDF]

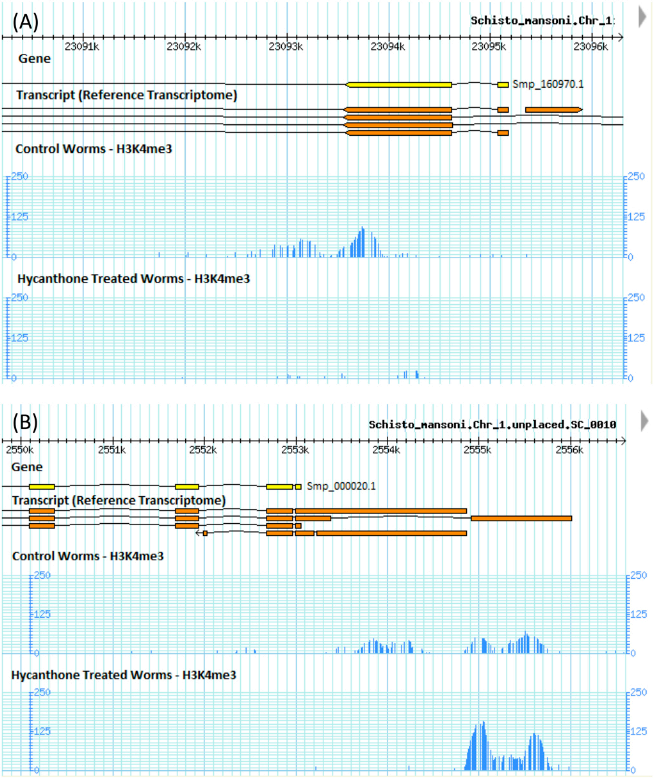

Supplementary Figure 1 : Two examples (A and B) of chromatin structure changes between control and hycanthone treated worms on the histone 3 tri-methylated on lysine 4 (H3K4me3) identified through the bioinformatic approach and followed by a visual inspection on our local genome browser. The gene track corresponds to the gene annotation from *Schistosoma mansoni* genome assembly v5.0 (Protasio et al., 2012) and the transcript track refers to a reference transcriptome including data from the genome assembly v5.0 and local RNA-seq data (unpublished).
